# Mental Health Problems, History of Drug Use, and Violent Offending Are Associated With Increased Suicide Risk in Imprisoned Females

**DOI:** 10.3389/fpsyt.2019.00395

**Published:** 2019-06-06

**Authors:** Shaoling Zhong, Xiaomin Zhu, Graham Mellsop, Huijuan Guo, Yanan Chen, Chenyuli Luo, Qiguang Li, Jiansong Zhou, Xiaoping Wang

**Affiliations:** ^1^Department of Psychiatry of the Second Xiangya Hospital, Mental Health Institute of Central South University, China National Clinical Research Center on Mental Disorders (Xiangya), China National Technology Institute on Mental Disorders, Hunan Key Laboratory of Psychiatry and Mental Health, Changsha, China; ^2^Suzhou Mental Health Center, Suzhou Guangji Hospital, the Affiliated Guangji Hospital of Soochow University, Suzhou, China; ^3^Waikato Clinical Campus, University of Auckland, Auckland, New Zealand

**Keywords:** suicide, female, prison, mental health, ridit analysis

## Abstract

**Background:** In western countries, imprisoned females are at high risk for suicide, but the risk in Chinese imprisoned females has not been well established. The aim of this study was to clarify the suicide risk and its correlates among imprisoned females in China.

**Methods:** In this cross-sectional study, subjects were recruited from the Female Prison of Hunan province, China. A standardized questionnaire was used to collect socio-demographic and criminological data. The Suicidality module of the Mini International Neuropsychiatric Interview (MINI) 5.0 and 12-item General Health Questionnaire (GHQ-12) were used to assess suicide risk and mental health problems, respectively. Ordinal logistic regressions were used to identify independent factors associated with increased suicide risk.

**Results:** A total of 2,709 imprisoned females completed the survey questionnaire. Twenty percent were rated as presenting suicide risk. Mental health problems [odds ratio (OR) = 1.21, 95% confidence interval (CI) = 1.00–1.47], self-reported help-seeking for mental health problems (OR = 1.69, 95% CI = 1.11–2.56), violent offending (OR = 1.69, 95% CI = 1.37–2.09), history of drug use (OR = 1.46, 95% CI = 1.15–1.84), family history of mental disorders (OR = 1.57, 95% CI = 1.10–2.23), marital status (OR = 1.29, 95% CI = 1.05–1.58), and low educational level (OR = 1.36, 95% CI = 1.11–1.67) were independently associated with increased suicide risk.

**Conclusion:** One fifth of the imprisoned females are at risk for suicide. This study highlights the importance of assessing mental health status for suicide prevention among female prisoners.

## Introduction

Worldwide, more than 10 million people are detained in prison (1). Imprisoned females generally account for approximately 7% of the total imprisoned population, and that figure is growing (2). The King’s International Centre for Prison Studies (ICPS) (www.prisonstudies.org) reported that the number of imprisoned females had reached 700,000 all over the world in 2015. In China, the total number of imprisoned females had increased by 38.8% over one decade (from 77,279 in 2005 to 107,237 in 2015), a growth rate about nine times that of males.

Existing evidence showed an increased suicide risk in prison settings, especially for female prisoners (3, 4). Compared with the general population, the suicide risk is increased ranged from 6- to 20-fold in female prisoners in western countries (3, 5). A recent meta-analysis including 24 high-income countries showed the suicide risk among females is around nine times than in the general population (6). Beyond these countries, little evidence represented suicide risk in low-income and middle-income countries, particularly in China. Previously effective assessment, management, and intervention to prevent suicides are important activities for policymakers, health services, and clinicians (7). In September 2018, the National Institute for Health and Care Excellence (NICE) released guidelines for suicide prevention in detention settings (8).

A number of dynamic risk factors have been identified that lead to elevated suicide risk in incarcerated settings. These include mental health needs (9) and mental health problems (10–13) or psychiatric morbidity, e.g., major depression (14, 15). Besides the high rate of mental health problems (16), the prison environment itself may also make females vulnerable to suicide. These modifiable risk factors would provide opportunities for further attention and intervention.

Similar to the general population (17, 18), there is evidence demonstrating that a quantity of fixed distal factors, including alcohol/drug misuse (19, 20) and early-life adversity (21), are well-established correlates for increased suicide risk. A family history of mental diseases, crime, and drug/alcohol misuse might also elevate the suicide risk by contributing to adverse childhood experiences (22). Although these suicide risk factors are far less useful in predicting individual’s risk, they might be beneficial in identifying high-risk groups (23).

Assessing suicide risk and providing relevant interventions are potentially cost-effective approaches to reducing suicides. Identified suicide risks appear to be sensitive predictors of actual suicide (24). Stratifying levels of suicide risk may facilitate the identification of relevant assessment, management, and intervention activities to improve outcomes in prison settings with limited mental healthcare service resources. The point has been made that the suicide risk factors in imprisoned females in China are poorly understood (25) as the available studies have been conducted in Western countries (14); therefore, we conducted this study to investigate suicide risk and related factors among imprisoned females in China. We hypothesize that certain socio-demographic, criminal, and mental health correlates are related to elevated suicide risks among imprisoned females.

## Methods

### Sample and Study Site

**Figure 1:**
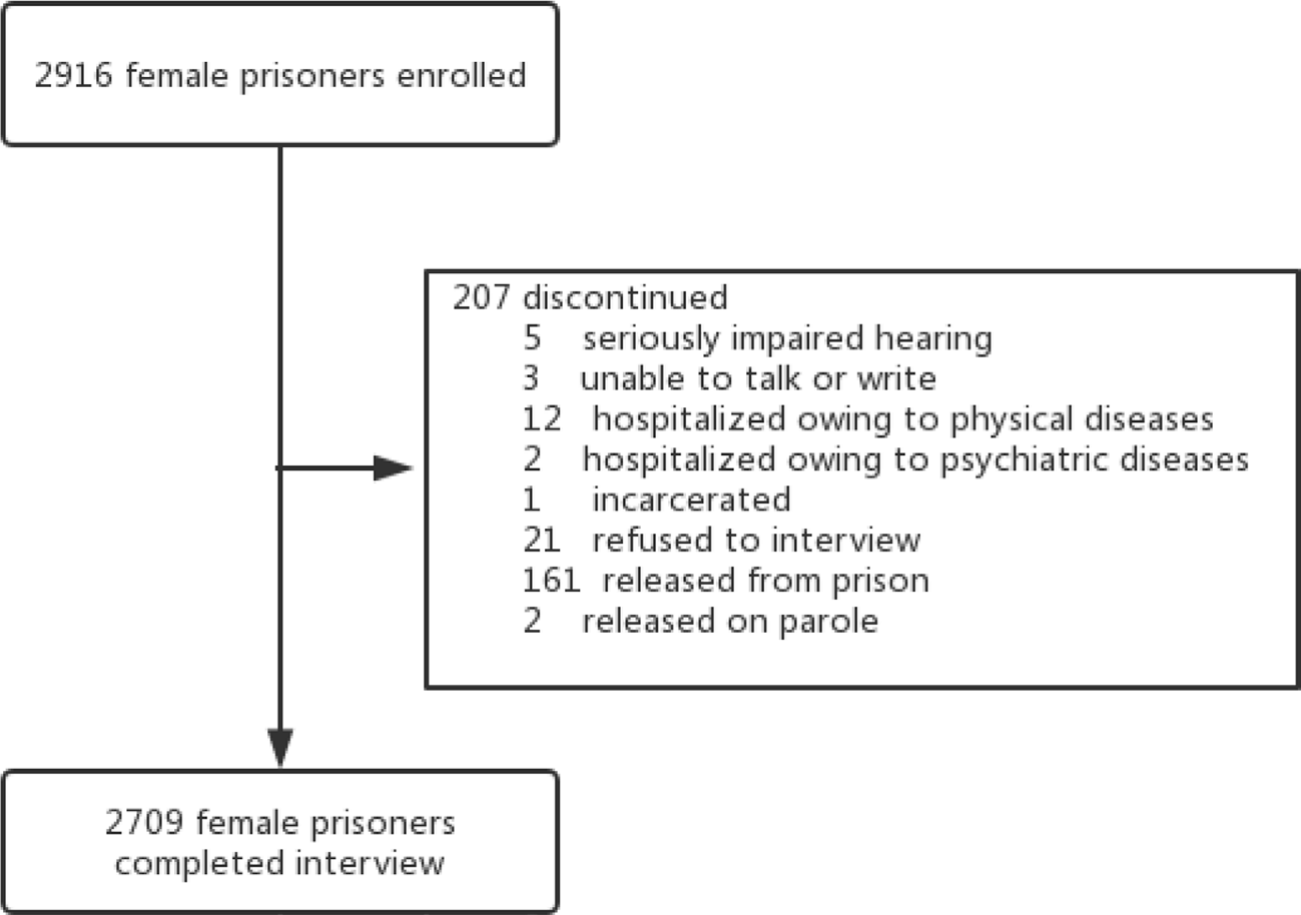
A sampling flow chart.

Subjects of this cross-sectional study were recruited from the only female prison in the Hunan province which covers nearly 70 million people in central-south China. The eligible criteria for the women were as follows: 1) Chinese nationality, 2) able to communicate with and comprehend the purpose of the survey, and 3) fluent in the Chinese language. The excluding criteria were as follows: 1) with severe physical illness and 2) refusal to participate. Participants who could not read or write but were willing to join completed the study with the help of an investigator. All participants were told that they were free to quit without any punishment. No reimbursement for participation was guaranteed. Anonymity and confidentiality were guaranteed prior to the study as well. Of the total 2,916 imprisoned females, 207 were not willing or not able to participate in the study (see **Figure 1**), leaving a total sample of 2,709 (92.9%). The study protocol was approved by the Clinical Research Ethics Committee of the Second Xiangya Hospital, Central South University, the authority of the Hunan Female Prison and the Bureau of Prisons in Hunan Province.

### Assessment Instruments

A purpose-designed form was used to collect socio-demographic and criminological data. The socio-demographics included age, education levels, marital status, residence, personal monthly income, employment prior to prison, smoking history, history of drug use, family history of mental disorders, family history of criminal crimes and family history of alcohol/drug use, seeking help for mental health problems, and previous hospitalization due to mental health problems. The history of drug use was assessed using a dichotomous item: “in your lifetime, have you ever used any of the following drugs more than once to get high, feel better, or to change your mood” (*The MINI-International Neuropsychiatric Interview, M.I.N.I*). The criminological factors including criminal history (including violent history), current violent offending, imprisonment duration, and sentence. The criminal history (including violent history) was administrated using a dichotomous item: Have you ever been convicted of a crime before. For those who answered “yes,” the type of crimes (homicide/assault/robbery/defraud/prostitution/drug trafficking etc.) was confirmed. A violent history was defined as homicide, assault, robbery, and arson. The crime history was further verified by the police records.

The 12-item General Health Questionnaire (26) (GHQ-12) was administrated to measure the mental health problems. It has been used in different settings, including prison (27, 28). The dichotomous scale (0, not at all, or same as usual; 1, rather more or much more than usual) of GHQ-12 items was used to assess whether participants have each symptom during the last 4 weeks. A threshold of 4 and above was used to indicate the presence of mental health problems (29). This tool has been introduced and validated in China previously with good reliability and validity (30).

*The MINI-International Neuropsychiatric Interview (M.I.N.I*), which is a short, structured interview tool for screening and diagnosing according to *Diagnostic and Statistical Manual of Mental Disorders, Fouth Edition* (DSM-IV) axis I, and 10th revision of the International Statistical Classification of Diseases and Related Health Problems (ICD-10), developed by Lecrubier and Sheehan (31), were administered to all participants. The Chinese version of M.I.N.I. was introduced by Mental Health center of Beijing University in 2006, with established reliability and validity (32, 33). Suicidality, the subscale of the M.I.N.I. 5.0, was administrated to assess suicide risk. There are six items in the tool as listed in **Table 1**. The points for the six items were added to get a total point score. In this study, four suicide risk levels were used: non-suicidal risk, low-suicidal risk, moderate-suicidal risk, and high-suicidal risk.

**Table 1:** Items of suicidality in the MINI-International Neuropsychiatric Interview (M.I.N.I.).

In the past month did you		Answers		Points
	think that you would be better off dead or wish you were dead?	No	Yes	1
	want to harm yourself or to injury yourself	No	Yes	2
	think about suicide	No	Yes	6
	have a suicide plan	No	Yes	10
	attempt suicide?	No	Yes	10
In your lifetime did you	make a suicide attempt?	No	Yes	4
Suicide risk*				

*The points for the six items are added to get a total point. The total point of 0 is ranked as non-risk, between 1–5 is ranked as low risk, 6–10 as moderate risk, and >10 as high risk.

### Procedures

Prior to the initiation of the survey, an advertisement about the purpose was distributed by the prison officers to all imprisoned females. After giving written informed consent, participants were asked to complete the standardized questionnaire with the assistance of our trained investigators (when necessary). Before collection of the questionnaire, our investigators checked the questionnaire to avoid missing data. After completing the questionnaire, participants were interviewed face-to-face individually in a quiet room at the prison by a trained psychiatrists, using the suicidality module of the M.I.N.I.

### Statistical Analyses

The IBM Statistic Package for Social Sciences (SPSS) version 18.0 was used for all statistical analyses. Categorical variables were presented as numbers and percentages, and continuous variables were presented as mean ± standard deviation (SD). To compare socio-demographic, criminological, and mental health factors across different suicidal risk groups, one-way analysis of variance (ANOVA) was used for continuous variables (i.e., age). Then, as the outcome is termed ordinal (non-/low-/moderate-/high-suicide risks), the Ridit analyses were adopted for all the other variables. The Ridit analyses, which consider the ordering of categories, were developed by Bross (34) and widely used in health care areas. Finally, the ordinal logistic regressions were performed to evaluate factors associated with increased or decreased suicide risks. Variables that were statistically significant on univariate analysis were entered into ordinal logistic regression. Two-tailed* p* values of 0.05 were set as statistically significant.

## Results

### Socio-Demographics, Criminological Characteristics, and Suicide Risk

The mean age of the 2,709 imprisoned females was 39.8 (SD = 10.4) years, ranging from 15 to 81 years. In the imprisoned females, 56.8% were married, 36.9% had received less than 6 years of education, 43.0% were unemployed prior to prison, and 6.8% had a criminal history.

A total of 548 (20.2%) reported apparent recent suicidal risk: 456 (16.8%) low, 78 (2.8%) moderate, and 14 (0.5%) high. The socio-demographic and criminological variables for the different suicidal risk groups are shown in **Table 2**. There are significant differences in age (*F* = 26.19, *p* < 0.01), education levels (*F* = 2.82, *p* = 0.04), employment (*F* = 5.09, *p* = 0.02) and marital status (*F* = 13.61, *p* < 0.01). Furthermore, violent offending (*F* = 25.81, *p* < 0.01), history of drug use (*F* = 8.68, *p* < 0.01), and family history of mental disorders (*F* = 12.21, *p* < 0.01) differed significantly between the four groups.

**Table 2:** Demographics and criminological factors among imprisoned females with increased suicide risk.

		All		Non-suicide		Low-suicidal risk		Moderate-suicidal risk		High-suicidal risk			F	*p* value
		*N* = 2,709		*n* = 2,161		*n* = 456		*n* = 78		*n* = 14				
		mean	s	median										
Age (years)		39.8	10.4	40.0	15.0	39.0	14.0	35.0	15.0	40.5	30.0		26.19	<0.01
		*n*	%	*n*	%	*n*	%	*n*	%	*n*	%	Ridit score		
Education^a^													2.82	0.04
	Low	1,000	36.9	770	35.6	195	42.8	29	37.2	6	42.9	0.66		
	High	1,709	63.1	1391	64.4	261	57.2	44	62.8	8	57.1	0.51		
Residence													1.13	0.29
	Urban	1,844	68.1	1483	68.6	292	64.0	61	78.2	8	57.1	0.60		
	Rural	865	31.9	678	31.4	164	36.0	17	21.8	6	42.9	0.58		
Employment													5.09	0.02
	Unemployed	1,166	43.0	908	42.0	208	45.6	44	56.4	6	42.9	0.62		
	Employed	1,543	57.0	1253	58.0	248	54.4	34	43.6	8	57.1	0.57		
Marriage													13.61	<0.01
	Married	1,539	56.8	972	45.0	163	35.7	28	35.9	7	50.0	0.56		
	Unmarried^b^	1,170	43.2	1189	55.0	293	64.3	50	64.1	7	50.0	0.61		
Monthly income^c^													2.29	0.10
	Low	67	2.5	55	2.5	11	2.4	1	1.3	0	0.0	0.40		
	Medium	1,642	60.6	1329	61.5	263	57.7	44	56.4	6	42.9	0.47		
	High	1,000	36.9	777	36.0	182	39.9	33	42.3	8	57.1	0.64		
Criminal history													0.07	0.80
	no	2,525	93.2	2015	93.0	427	93.6	72	92.3	11	78.6	0.60		
	yes	184	6.8	146	6.8	29	6.4	6	7.7	3	21.4	0.52		
Violent offending													25.81	<0.01
	no	1,861	68.7	1535	71.0	271	59.4	47	60.3	9	64.3	0.58		
	yes	848	31.3	627	29.0	185	40.6	31	39.7	5	35.7	0.63		
Smoking													1.64	0.20
	no	1,975	72.9	1587	73.4	325	71.3	53	67.9	10	71.4	0.56		
	yes	734	27.1	575	26.6	131	28.7	25	32.1	4	28.6	0.66		
History of drug use													8.68	<0.01
	Never	2,008	74.1	1626	75.2	332	72.8	42	53.8	8	57.1	0.53		
	Ever	701	25.9	535	24.8	124	27.2	36	46.2	6	42.9	0.73		
Family history of drug use													0.05	0.82
	no	2,502	92.4	1995	92.3	420	92.1	73	93.60	14	100.0	0.59		
	yes	207	7.6	166	7.7	36	7.9	5	6.40	0	0.0	0.71		
Family history of alcohol													1.60	0.21
	no	986	36.4	801	37.1	146	32.0	31	39.7	8	57.1	0.55		
	yes	1,723	63.6	1360	62.9	310	68.0	47	60.3	6	42.9	0.61		
Family history of crime													1.05	0.31
	no	2,332	86.1	1869	86.5	379	83.1	70	89.7	14	100.0	0.60		
	yes	377	13.9	292	13.5	77	16.9	8	10.3	0	0.0	0.55		
Family history of mental diseases													12.21	<0.01
	no	2,546	94.0	2048	94.8	416	91.2	71	91.0	11	78.6	0.59		
	yes	163	6.0	113	5.2	40	8.8	7	9.0	3	21.4	0.61		
Sentence duration (months)													1.47	0.21
	0–12	33	1.2	27	1.2	4	0.9	2	2.6	0	0.0	0.69		
	13–60	733	27.1	603	27.9	96	21.1	27	34.6	7	50.0	0.63		
	61–120	620	22.9	503	23.3	100	21.9	13	16.7	4	28.6	0.55		
	121–240	589	21.7	458	21.2	116	25.4	14	17.9	1	7.1	0.57		
	life sentence	734	27.1	570	26.4	140	30.7	22	28.2	2	14.3	0.61		
Imprisonment duration													2.294	0.10
	1–12 months	347	12.8	276	12.8	59	12.9	11	14.1	1	7.1	0.60		
	13–60 months	1,395	51.5	1134	52.5	212	46.5	40	51.3	9	64.3	0.60		
	61–120 months	636	23.5	502	23.2	111	24.3	20	25.6	3	21.4	0.53		
	>120 months	331	12.2	249	11.5	74	16.2	7	9.0	1	7.1	0.74		
Seeking help													12.96	<0.01
	no	2582	95.3	2076	96.1	419	91.9	75	96.2	12	85.7	0.59		
	yes	127	4.7	85	3.9	37	8.1	3	3.9	2	14.3	0.56		
Hospitalization history													6.11	0.01
	no	2673	98.7	2138	98.9	446	97.8	77	98.7	12	85.7	0.60		
	yes	36	1.3	23	1.1	10	2.2	1	1.3	2	14.3	0.59		
Mental health problems													5.36	0.02
	no	1718	63.4	1394	64.5	269	59.0	46	59.0	9	64.3	0.59		
	yes	991	36.6	767	35.5	187	41.0	32	41.0	5	35.7	0.60		

a Education level ≤6 years was defined as low, education level >6 years was defined as high.

b Unmarried includes single, divorced, and widow.

c Monthly Income ≤ poverty threshold (¥2,300 per person per year in 2013 in China) was defined as low, poverty threshold <monthly income ≤ per capita disposable income *(*¥23,414 per person per year in 2013 in Hunan*)* was defined as medium, monthly income > per capital disposable income was defined as high.

### Comparison of Mental Health Status Between the Different Suicidal Risk Groups

**Table 2** also shows the mental health status among the four groups. A total of 991 (36.6%) imprisoned females reported the presence of mental health problems. One hundred twenty-seven (4.7%) of them had ever sought help for their mental health problems, with only 28.3% (36/127) having experienced hospitalization. The presence of mental health problems (*F* = 5.36, *p* = 0.02), seeking help for mental health problems (*F* = 12.96, *p* < 0.01), and hospitalization history due to mental health problems (*F* = 6.11, *p* = 0.01) were all significantly related to increased suicidal risk.

### Correlates of Suicide Risk

As shown in **Table 3**, several factors were significantly associated with increased suicide risk. These factors were violent offending [odds ratio (OR) = 1.69, 95% confidence interval (CI) = 1.37–2.09], history of drug use (OR = 1.46, 95% CI = 1.15–1.84), family history of mental disorders (OR = 1.57, 95% CI = 1.10–2.56), seeking help for mental health problems (OR = 1.69, 95% CI = 1.11–2.56), married status (OR = 1.29, 95% CI = 1.05–1.58) and low education level (OR = 1.36, 95% CI = 1.11–1.67).

**Table 3:** Ordinal logistic regression model assessing variables associated with increased suicide risk.

Variables		OR	95% CI		*p* value
			Lower	Upper	
Age		0.99	0.98	1.00	0.02
Marital status	Married	1.29	1.05	1.58	0.02
	Unmarried^a^	1.00			
Unemployment		1.12	0.91	1.37	0.29
Education^b^	Low	1.36	1.11	1.67	<0.01
	High	1.00			
Violent offending		1.69	1.37	2.09	<0.01
History of drug use		1.46	1.15	1.84	<0.01
Family history of mental disorders		1.57	1.10	2.23	0.01
Seeking help		1.69	1.11	2.56	0.01
Hospitalization history		1.53	0.73	3.21	0.26
Mental health problems		1.21	1.00	1.47	0.05

OR, odds ratio; 95% CI, 95% confidence interval.

a Unmarried status includes single, divorced and widow.

b Education level ≤6 years was defined as low; education level >6 years was defined as high.

## Discussion

To our knowledge, this is the first study to examine suicide risk and its socio-demographic, criminal, and mental health correlates among imprisoned females in China. This study revealed that one fifth of imprisoned females demonstrated recent suicide risk. Mental health problems, seeking help for mental health problems prior to the prison, family history of mental disorders, history of drug use, violent offending, being married, and a lower education level were associated with increased suicide risk. These findings provide clinically relevant information for identifying high-risk individuals and underpin the importance of screening for mental health problems and providing adequate psychiatric care in female prison.

The 20.2% prevalence of suicide risk in Chinese imprisoned females is comparable with the rate demonstrated in imprisoned females of French Guiana (20.0%) (35). Among the general population, female gender per se is an independent risk factor of suicidality (36). The complex, unmet female-sensitive needs in prisons might explain the high suicide risk in imprisoned females (37).

As described in western countries (10, 12), mental health problems are significantly associated with increased suicide risk. As mental health service is important for suicide prevention (18, 38), identifying and treating persons with mental health problems should be considered a routine component of mental health service. Earlier studies have found that the presence of family history of mental disorders is independently associated with increased suicide risk both in the community (39, 40) and prison settings (41), which we confirm. In addition to standard screening tools, our findings supported that self-reported help-seeking for mental health problems prior to the imprisonment is another correlate of suicide risk. This probably reflects that a number of imprisoned females with suicide risk may have been treated for their mental health problems prior to going in to prison (42). This finding is partly consistent with the NICE guideline, which emphasizes the importance of assessing mental health status and help-seeking behaviors in detention settings to identify people at high risk. Therefore, one implication for these is to screen for mental health problems and history of seeking help for mental health problems. It would also follow that it is likely to be helpful to reduce suicide risk to encourage help-seeking behaviors in the prison and to provide useful information on how and where to seek help.

High prevalence of drug use has been consistently reported in prison populations. This has been shown to be particularly the case for females (19, 43). A systematic review (43) reported that the prevalence of drug abuse or dependence varied from 30% to 60% in imprisoned females in western countries. The prevalence of drug use (15.9%) in our study is substantially lower than those figures, which could be ascribed to cultural and legal system differences between China and western countries. History of drug use has been frequently reported to be associated with both suicide risk and completed suicides (44, 45) in prison population. In line with these findings, drug use was associated with elevated suicide risk in this study. This relationship could be due to the withdrawal symptoms (both acute and prolonged) of these drug abusers, including depression, anxiety, and pain. Another possible explanation for this is that impulsiveness might work as a mediation between drug use and suicide risk. Earlier studies found that prison inmates with a lifetime history of drug use and suicide risk often have higher impulsiveness (46–48). Further pathway studies looking specifically at impulsiveness could improve understanding relationships between drug use and suicide risk. The robust relation between drug use and suicide risk suggest the importance of targeting those imprisoned females with history of drug use for further evaluation and management to reduce their risk of suicide.

A strong correlation between criminal characteristics and suicide risk has been previously reported in a systematic review (10). Imprisoned females charged with violent offending demonstrated elevated suicide risk in our study, replicating findings in other countries (42, 44, 45, 49). This may be contributed to by to shame, guilt, and stigma associated with violent offending. Previous researchers have suggested sentence duration is associated with increased suicide risk (36, 50), in particular lifetime sentences (10). In this study, no such association was found. Further studies about specific offense type and sentence duration are necessary to clarify these apparent discrepancies. Given the criminological influences in imprisoned females, additional attention and interventions for those with certain criminal characteristics should also be given priority.

## Implications

There are two main implications. First, the reported prevalence of suicide risk underlines the need to assess suicide risk among incarcerated females, despite the 3% of them having a moderate-to-high suicide risk. The second implication is that the findings support the hypothesis that suicide risks in incarcerated females are relevant to detective correlators. Although these risk factors might not be effective to predict suicide, they could be identified to assess suicide risk for the stakeholders. In practice, the presence of low suicide risk would need a dynamic assessment, while those with high suicide risk could lead to a further clinical examination by mental health professionals.

## Limitations

Some limitations should be clarified. First, the retrospective and cross-sectional study designs have the inability to draw a causal or pathogenic relationship between identified correlates and increased suicide risk. So, whether these factors result in an increase in suicide risk needs to be verified in prospective studies. Also, the self-report nature of the data could also add potential bias due to sensitivity and stigma of suicide (51). Second, suicide risk of imprisoned females might be underestimated, because this study was conducted in prison, and participants may conceal or deny their real intention to die. Third, due to limited interview time, this study did not collect data on other potentially important risk factors of suicide, such as childhood trauma (52).

## Conclusion

In conclusion, suicidal risk in 20.2% of imprisoned females was measured in China. Independent associated factors included mental health problems, family history of mental disorders, history of drug use, violent offending, marital status, and education level. These findings may facilitate targeting management, effective intervention with limited sources, and contribution to prevention. Further longitudinal follow-up would help determine the value of these factors in predicting suicide and in informing the development of preventive strategies.

## Data Availability Statement

The datasets generated for this study are available on request to the corresponding author.

## Ethics Statement

The study protocol was approved by the Clinical Research Ethics Committee of the Second Xiangya Hospital, Central South University, the authority of the Hunan Female Prison and the Bureau of Prisons in Hunan Province.

## Author Contributions

All authors have been involved in the preparation and completion of the study. They have also read and approved this report.

## Funding

This work was supported and funded by the National Key Research and Development Program of China (2016YFC0800701), National Natural Science Foundation of China [grant number: 81571341], [grant 81371500], National Key Research and Development Program of Hunan province [grant: 2018SK2133] and Natural Science Foundation of Hunan, China [grant: 2019SK0511]. National Natural Science Foundation of China (81571316) and the Natural Science Foundation of Jiangsu Province (BK20180213).

## Conflicts of Interest Statement

The authors declare that the research was conducted in the absence of any commercial or financial relationships that could be construed as a potential conflict of interest.
